# Nomograms integrating CT radiomic and deep learning signatures to predict overall survival and progression-free survival in NSCLC patients treated with chemotherapy

**DOI:** 10.1186/s40644-023-00620-4

**Published:** 2023-10-22

**Authors:** Runsheng Chang, Shouliang Qi, Yanan Wu, Yong Yue, Xiaoye Zhang, Wei Qian

**Affiliations:** 1 College of Medicine and Biological Information Engineering, Northeastern University, Shenyang, China; 2 Key Laboratory of Intelligent Computing in Medical Image, Ministry of Education, Northeastern University, Shenyang, China; 3 Department of Radiology, Shengjing Hospital of China Medical University, Shenyang, China; 4 Department of Oncology, Shengjing Hospital of China Medical University, Shenyang, China

**Keywords:** Nomogram, Overall survival, Progression-free survival, Lung cancer, Chemotherapy treatment, Radiomics

## Abstract

**Objectives:**

This study aims to establish nomograms to accurately predict the overall survival (OS) and progression-free survival (PFS) in patients with non-small cell lung cancer (NSCLC) who received chemotherapy alone as the first-line treatment.

**Materials and methods:**

In a training cohort of 121 NSCLC patients, radiomic features were extracted, selected from intra- and peri-tumoral regions, and used to build signatures (S1 and S2) using a Cox regression model. Deep learning features were obtained from three convolutional neural networks and utilized to build signatures (S3, S4, and S5) that were stratified into over- and under-expression subgroups for survival risk using X-tile. After univariate and multivariate Cox regression analyses, a nomogram incorporating the tumor, node, and metastasis (TNM) stages, radiomic signature, and deep learning signature was established to predict OS and PFS, respectively. The performance was validated using an independent cohort (61 patients).

**Results:**

TNM stages, S2 and S3 were identified as the significant prognosis factors for both OS and PFS; S2 (OS: (HR (95%), 2.26 (1.40–3.67); PFS: (HR (95%), 2.23 (1.36–3.65)) demonstrated the best ability in discriminating patients with over- and under-expression. For the OS nomogram, the C-index (95% CI) was 0.74 (0.70–0.79) and 0.72 (0.67–0.78) in the training and validation cohorts, respectively; for the PFS nomogram, the C-index (95% CI) was 0.71 (0.68–0.81) and 0.72 (0.66–0.79). The calibration curves for the 3- and 5-year OS and PFS were in acceptable agreement between the predicted and observed survival. The established nomogram presented a higher overall net benefit than the TNM stage for predicting both OS and PFS.

**Conclusion:**

By integrating the TNM stage, CT radiomic signature, and deep learning signatures, the established nomograms can predict the individual prognosis of NSCLC patients who received chemotherapy. The integrated nomogram has the potential to improve the individualized treatment and precise management of NSCLC patients.

**Supplementary Information:**

The online version contains supplementary material available at 10.1186/s40644-023-00620-4.

## Highlights

Integrated nomograms aim to predict the chemotherapy prognosis of NSCLC patients.

The 3- and 5-year overall survival and progression-free survival are predicted.

CT peritumoral radiomic signature has the highest hazard ratio for the prognosis.

Deep learning signature is a significant predictive factor of the prognosis.

The integrated nomograms are noninvasive, low cost, and phenotypic.

**Supplementary Information:**

The online version contains supplementary material available at 10.1186/s40644-023-00620-4.

## Introduction

Lung cancer presents the highest global mortality among cancer-related diseases in both men and women and remains one of the most common malignancies [1, 2]. Non-small-cell lung cancer (NSCLC) accounts for approximately 85% of all lung cancer cases, and its prevalence continues to increase [3]. The 3-year survival rate of NSCLC patients is clinically less than 30%, with a median survival time of 8–9 months [4, 5].

Chemotherapy is the most widely used first-line of treatment (monotherapy or combined therapy) for NSCLC with various prognoses [6, 7], which are affected by clinical pathology factors such as age, sex, smoking status, metastatic, pathological and genotype [8]. Accurately predicting the chemotherapy response for each patient with NSCLC may benefit the decision-making of the treatment plan and overall survival (OS).

The Tumor, Node, Metastasis (TNM) staging system is a standard of disease staging and prognosis prediction endorsed widely by the American Joint Committee on Cancer (AJCC) and the Union for International Cancer Control (UICC). It is important to note that we implemented the seventh edition of this system. Introduced in 2010, this edition has been broadly adopted since. In the seventh edition of the TNM system, ‘T’ refers to the size and local invasiveness of the primary tumor, ‘N’ indicates whether the lymph nodes are involved, and ‘M’ provides information on metastasis. Each category is further staged from 0 (none or minimum) up to the highest level (most severe or extensive). By considering these variables, the overall stage of a patient’s disease is determined, which is instrumental in estimating the recurrence and survival rates. [9]. However, the prognosis of patients with the same stage varies widely because this system only considers three anatomic features while overlooking several significant characteristics, such as smoking status and radiomic features [10, 11]. Ou et al. analyzed the smoking status of NSCLC patients and found that it was a favorable prognostic factor for OS [12]. Song et al. extracted the intensity, shape, and texture features of EGFR-mutated NSCLC patients and successfully predicted their progression-free survival (PFS).

A nomogram is a common tool for predicting the prognosis of patients [13] and can provide a numerical probability of clinical endpoints by creating an intuitive graph. Song et al. established a nomogram to predict PFS after EGFR tyrosine kinase inhibitor therapy in NSCLC patients to improve personalized management [14]. Deng et al. constructed a nomogram to predict the individual prognosis of NSCLC patients with distant metastasis and identified alternative several prognostic factors [15]. Liang et al. developed a nomogram to predict the survival of patients with resected NSCLC [16]. However, few studies have focused on predicting the prognosis of patients with NSCLC treated only with chemotherapy as the first-line treatment.

Moreover, the current studies on fitting survival curves with nomogram mainly applied clinical information combined with radiomic features or deep learning features [17]. Yang et al. developed a radiomics nomogram by combining 2D and 3D radiomics features and clinical predictors to assess the OS of NSCLC patients [18]. Tian et al. used a deep learning model to obtain PD-L1 expression signature and combined it with clinical models to predict immunotherapy response [19]. Multimodal models can leverage the complementary information from various sources (such as CT imaging, pathological features, clinical data, etc.) and various feature extraction models (like radiomics, deep models, etc.) to enhance the comprehensive predictive capabilities of the model for tasks such as tissue segmentation, biomarker discovery, and clinical prognosis [17, 20]. This approach also better understands patient-specific characteristics, tissue heterogeneity, and disease progression in tumors, thereby providing more precise and comprehensive predictions [21–23].

This research aimed to combine information from different modalities (clinical information, radiomic features (intratumor and peritumor) [20]and deep learning features) to fit the survival curves of patients. Furthermore, we developed nomograms for predicting OS and PFS after first-line chemotherapy in patients with NSCLC.

## Materials and methods

### Study design

**Fig. 1:**
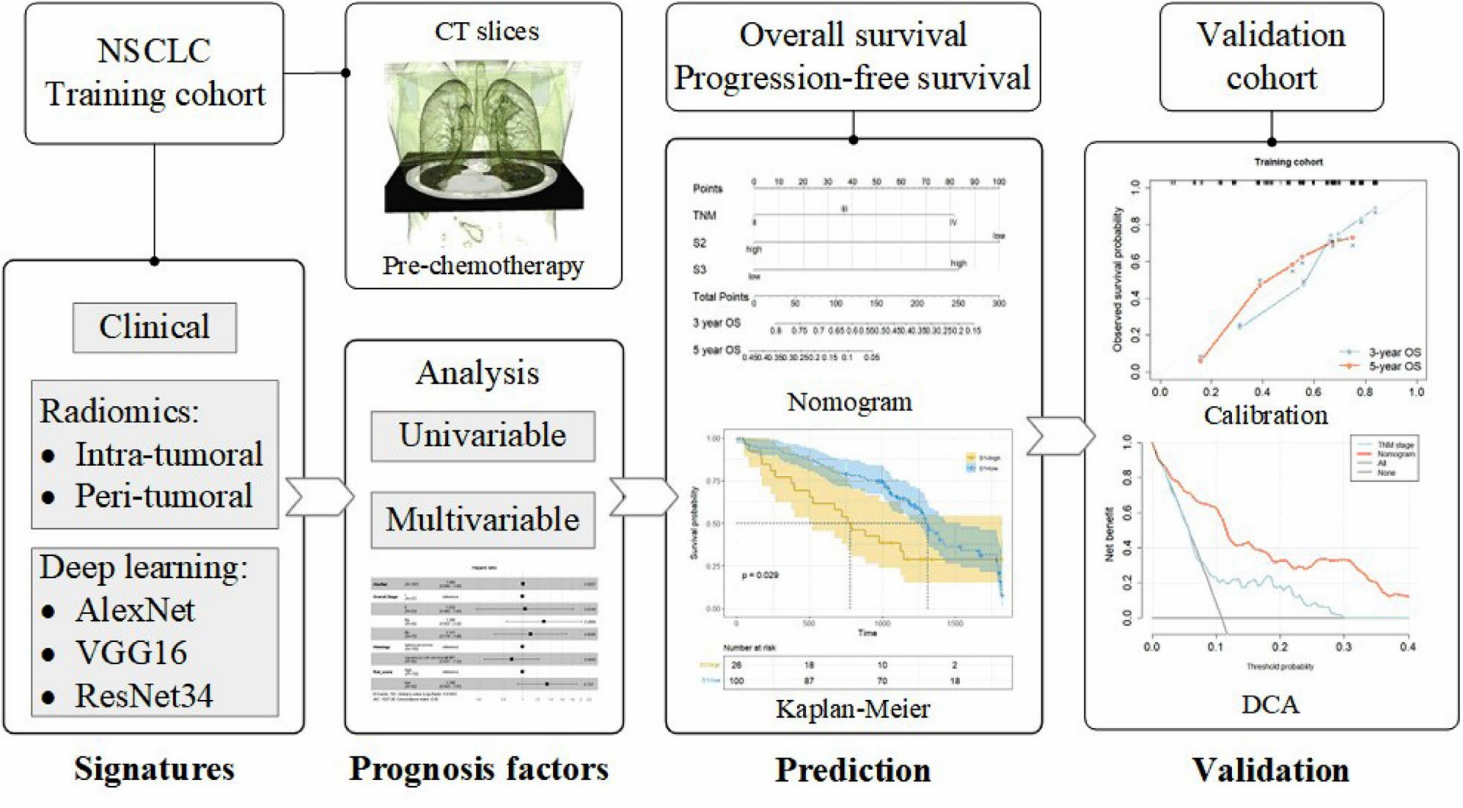
Flowchart of the study

A flowchart of the study is presented in Fig. 1. Clinical, radiomics, and deep learning features were first extracted to identify the characteristics of each pre-chemotherapy patient. Signatures for these features were then proposed using the least absolute shrinkage and selection operator (LASSO) Cox proportional hazards regression method to stratify the risk of prognosis in the training cohort. This was followed by analyzing the phenotypic signatures using univariate and multivariate Cox regression, and nomograms were established for OS and PFS prediction. Finally, the results were validated using calibration blots and decision curve analysis (DCA) in an independent validation cohort.

### Patients

The institutional review board of the Shengjing Hospital of China Medical University approved this retrospective study and waived the need for informed consent from patients. A total of 187 patients (from 605) with NSCLC were retrospectively enrolled from the Shengjing Hospital of China Medical University between 2016 and 2021 for this study. Only patients who were treated with first-line chemotherapy were included, and those with a history of surgical resection, radiology, and targeted medicine or immunity therapy were excluded. Patients who lacked CT images before and after chemotherapy, diagnostic reports, or follow-up records were excluded from the study.

We assessed the treatment response according to the response evaluation criteria in solid tumors 1.1 [24] standard and diagnosed reports every 12 weeks until disease progression, death, or withdrawal from this study. The endpoints of this study were OS and PFS; OS was defined as the time from the initiation of chemotherapy to the date of death, and live patients were censored at the last follow-up. PFS was defined as the time from the initiation of chemotherapy to the date of recurrence (development of a new lesion or progression of the primary lesion) or death. Patients alive without recurrence were censored at the last follow-up. Clinical characteristics such as sex, age, histological type, pathologic stage, tumor location, and smoking history were recorded for all eligible patients. The pathological stage was characterized according to the TNM staging system. Eligible patients were randomly divided into 2:1 training (n = 126) and validation (n = 61) groups.

### Segmentation, radiomic feature extraction, selection, and signature building

All pre-chemotherapy CT image voxels were interpolated to 1 × 1 × 1 mm to eliminate the influence of varying acquisition equipment. The primary tumors of all the patients were semi-automatically segmented by manually labeling the seed points and then applying the seed growth algorithm of the 3D slicer software [25] for segmentation, which was then manually corrected under the guidance of two radiologists with more than 20 years of experience.

Both radiologists were blinded for the entire segmentation procedure. The Dice coefficient and over- and under-lesion segmentation errors were calculated to determine the inter-observer agreement. Based on the segmented intra-region of the tumor, a 3D morphological dilation operation was performed with three pixels. After subtracting the intratumoral region, a 0–3 mm peritumoral region was obtained (We used the same segmentation procedure as our previous study [26], but the data was different).

A total of 1688 radiomic features, including first-order, shape, and texture features, were extracted from the tumor region using the open-source PyRadiomics Python package [27]. All features extracted from original and derived images (LoG with 5 sigma levels, 1 level of Wavelet decomposistions yielding 8 derived images and images derived using Square, Square Root, Logarithm and Exponential filters). We used the LASSO [28] penalized Cox proportional hazards regression to screen the significant features and calculate their corresponding weights for a treatment response from all the extracted features in the training cohort.

Signatures of the intratumoral (S1) and peri-tumoral (S2) regions were built for each patient using the weighted linear combination of all the significant features. Using the X-tile plot based on the Kaplan-Meier survival analyses and log-rank test [29], the signature was applied to stratify the training cohort into over- and under-expression progression subgroups of the chemotherapy response. The X-tile [30] provides the optimal binary threshold of the signature for risk stratification to enable the comparison of different prognostic factors in stratified subgroups.

### Deep learning features extraction and signature building

A 64 × 64 × 32 cuboid containing an entire tumor was cropped from pre-chemotherapy CT images for each patient by two experienced radiologists. Each slice was resized to adapt to different models and converted to an RGB format (make three copies of each slice and overlay them to simulate RGB data in 3D format). We extracted the features using three pre-trained backbone modules (AlexNet, VGG16, and ResNet34). The backbone network in this study was pre-trained using images from ImageNet [31] and fine-tuned using CT slices. The output of the three backbone networks was a vector with the following dimensions: 32 × 7 × 7 × 256, 32 × 7 × 7 × 512, and 32 × 7 × 7 × 512. Subsequently, a global average pooling layer (7 × 7) and a maximum pooling layer (32) were used to obtain a 256/512-dimensional vector (Supplemental Fig. S1**)**. For nomogram construction and clinical utility, we transformed the deep learning features arithmetically into a quantifiable measure, allowing us to conduct survival curve analysis on patients. The result was then employed to stratify patients into high-risk and low-risk cohorts, enabling a visual representation of patient risk in clinical settings. The signature was built by passing the vector through a fully connected layer to a neuron with a linear activation function. We used X-tile to obtain the optimal cut-off threshold to stratify the training cohort into over- and under-expression subgroups following chemotherapy.

### Statistical analysis

We applied nomograms to fit the survival curves of the patients in three steps: (1) The prognostic risks of OS and PFS in the training cohort were identified using the unadjusted univariable Cox regression analysis. (2) Variables that reached a statistical significance (p < 0.05) were included in the Cox proportional hazards multivariable regression [32] by calculating the hazard ratios (HRs) and 95% CIs. (3) According to the results of the Cox proportional hazards model, variables that remained statistically significant (p < 0.05) were incorporated to construct the nomogram. In this study, the input includes six clinical characteristics, two radiomics features (S1, S2) and three deep learning features (S3, S4 and S5). After statistical analysis, the nomogram models integrated all predictive features to fit survival curves.

We used the Kaplan-Meier (KM) method to generate survival curves to represent OS and PFS and evaluated between-group comparisons using a log-rank test that was stratified for the signatures of significant features.

The nomogram adopted the 3- and 5-year OS and PFS rates as the endpoints. We used Harrell’s concordance index (C-index) with a 95% CI to evaluate the discriminative ability of the nomogram using 1,000 bootstrap resamples for the internal validation in the training and validation cohorts, respectively. Calibration blots in the two sets were developed to visualize the agreement between the predicted and observed 3- and 5-year OS and PFS rates to assess the predictive accuracy of the nomogram. Finally, the performance of our model was compared with that of the TNM staging system using DCA. All statistical analyses were performed using R software (version 3.3.3, http://www.Rproject.org).

## Results

### Patients and clinicopathologic features

A total of 187 patients with NSCLC were enrolled in this study according to the inclusion and exclusion criteria. The demographic and histopathological characteristics of the enrolled patients are shown in Table 1. All patients were randomly allocated into two parts: 126 patients (mean (SD) age, 62.43 (12.15) years; median age, 55 years; 67 (53.2%) female) in the training cohort, and 61 patients (mean (SD) age, 60.74 (13.25) years; median age, 50 years; 35 (57.4%) female) in the validation cohort. The median follow-up period was 28.3 (0–60.0) and 25.6 (5.8–55.0) months in the training and validation cohorts, respectively. No significant survival difference in OS and PFS was found among the training cohort (OS: median, 36.7 months; PFS: median, 24.2 months) and the validation cohort (OS: median, 32.5 months; PFS: median, 21.8 months). In addition, no significant statistical differences (p > 0.05) were found in the demographic characteristics (sex, age, smoking status, histopathology, tumor location, and TNM stage) among the two cohorts.

**Table 1:** Demographic and histopathologic characteristics of study patients

Characteristic	Patients, No. (%)			
	Training cohort	P*	Validation cohort	P*
Sex		1.05		1.24
Male	59 (46.8)		26 (42.6)	
Female	67 (53.2)		35 (57.4)	
Age, median (range), y	55 (32–83)	1.36	50 (37–87)	1.02
Smoking status		0.95		0.84
Ever	71 (56.3)		33 (54.1)	
Never	55 (43.7)		28 (45.9)	
Histopathology		0.16		0.08
ADC	108 (85.7)		51 (83.6)	
SCC	18 (14.3)		10 (16.4)	
Tumor location		0.23		0.34
Left	67 (53.2)		34 (55.7)	
Right	46 (36.5)		21 (34.4)	
Others	13 (10.3)		6 (9.9)	
TNM stage		< 0.001		< 0.001
II	16 (12.7)		9 (14.8)	
III	78 (61.9)		39 (63.9)	
IV	32 (25.4)		13 (21.3)	

Adenocarcinoma (ADC), squamous cell carcinoma (SCC).

*P-value: it is calculated by two-sample t-test for the comparison between two groups categorized by high or low risk of S2

### Radiomic features and signatures

Phenotypic features were extracted from the intra- and peritumoral regions of the CT images of each patient acquired before chemotherapy. For the inter-observer reproducibility of segmentation by the two radiologists, the Dice coefficient was 0.86 ± 0.04, and the over- and under-segmentation errors of the segmented tumor volume were 0.19 ± 0.11, 0.26 ± 0.11, respectively.

In the training cohort, 1688 radiomic features were obtained from the intra- and peritumoral regions of each patient. Subsequently, 12 (intratumoral) and 9 (peritumoral) significant features were screened using the LASSO Cox proportional hazards regression model. The weights of the 12 selected features were used to build the signature (S1) of the intratumoral region, and the weights of the nine selected features were used to build the signature (S2) of the peritumoral region. Based on S1 and S2, a cutoff threshold of 0.27 and 0.70 were respectively obtained by X-tile with the maximum Chi-squared log-rank value to stratify the NSCLC patients into over-expression and under-expression groups.

### Deep learning features and signatures

Deep learning features were extracted from each pretrained model (AlexNet, VGG16, and ResNet34) in the training cohort. Following the backbone module, a global average pooling layer, max pooling layer, and fully connected layer were used to obtain the signatures (S3, S4, and S5). Each signature was then calculated using X-tile to stratify the NSCLC patients into over-expression and under-expression groups; the cutoff values of S3, S4, and S5 were − 0.83, 0.33, and 0.62, respectively.

### Independent prognostic factors in the training set

For both OS and PFS, a univariable unadjusted Cox analysis was performed for the following factors: sex, age, smoking status, histopathology, tumor location, TNM stage, S1, S2, S3, S4, and S5 in the training cohort. In OS, TNM stage (HR (95%): III, 1.49 (1.03–2.21), p < 0.05; IV, 2.28 (1.19–4.36), p < 0.05), S1 (HR (95%), 0.56 (0.33–0.95); p < 0.05), S2 (HR (95%), 2.48 (1.56–3.94); p < 0.001), and S3 (HR (95%), 0.43 (0.26–0.69) p < 0.001), were identified as statistically significant prognostic features. In PFS, TNM stage (HR (95%): III, 1.45 (1.03–2.38), p = 0.14; IV, 2.15 (1.13–4.13), p < 0.05), S1 (HR (95%), 0.58 (0.34–0.99), p < 0.05), S2 (HR (95%), 2.45 (1.53–3.94), p < 0.001), and S3 (HR (95%), 0.45 (0.28–0.74), p < 0.05), were identified as statistically significant prognostic features.

**Fig. 2:**
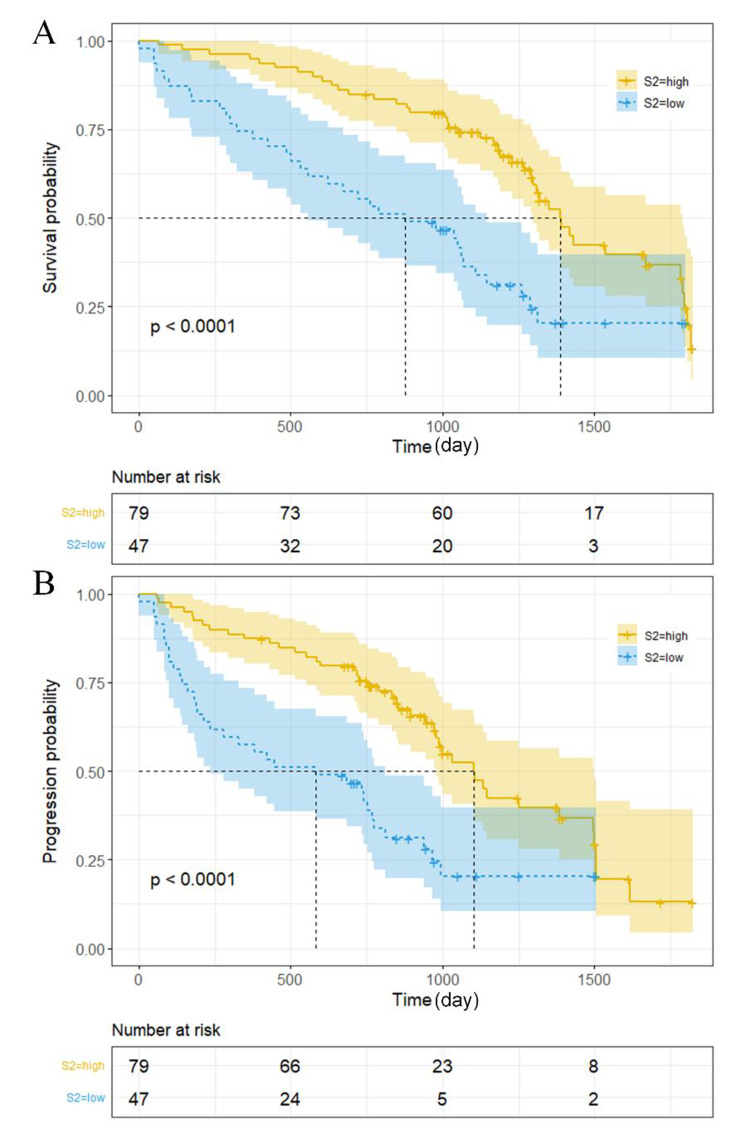
Kaplan-Meier curves of S2 for over- and under-expression subgroups: (**A**) Kaplan-Meier curves in OS; (**B**) Kaplan-Meier curves in PFS.

We analyzed the over- and under-expression subgroups of OS and PFS using Kaplan-Meier curves; Fig. 2A and B confirm the significant differences in both OS and PFS of S2 between the two groups. In OS, the subgroups with the under-expression (median, 29.2 months) signatures tend to have a lower survival probability than those with over-expression (median, 45.7 months) (p < 0.001). Similar to PFS, the subgroups with under-expression (median, 20.3 months) signatures tend to have a lower survival probability than over-expression (median, 36.7 months) (p < 0.001). The KM curves for TNM, S1, and S3 are shown in Supplemental Fig. S2, Fig. S3 and Fig. S4.

These prognostic features were included in the Cox multivariate analysis; the results are shown in Table 2. Apparently, TNM stage (HR (95%): III, 1.48 (1.04–2.47), p = 0.13; IV, 2.10(1.09–4.04), p < 0.05), S2 (HR (95%), 2.26 (1.40–3.67), p < 0.001), and S3 (HR (95%), 0.48 (0.29–0.79), p < 0.05) remained significantly associated with OS. Similarly, TNM stage (HR (95%): III, 1.42 (1.06–2.34), p = 0.17; IV, 1.98(1.02–3.84), p < 0.05), S2 (HR (95%), 2.23 (1.36–3.65); p < 0.05), and S3 (HR (95%), 0.55 (0.33–0.90); p < 0.05) were independently associated with PFS.

**Table 2:** Results of multiple cox regression

Subgroup	Patients, No.	OS		PFS	
		HR (95% CI)	P	HR (95% CI)	P
TNM stage					
II	65	Reference		Reference	
III	42	1.48 (1.04–2.47)	0.13	1.42 (1.06–2.34)	0.17
IV	19	2.10 (1.09–4.04)	< 0.05	1.98 (1.02–3.84)	< 0.05
S2			< 0.001		< 0.05
Over-expression	79	Reference		Reference	
Under-expression	47	2.26 (1.40–3.67)		2.23 (1.36–3.65)	
S3			< 0.05		< 0.05
Over-expression	92	Reference		Reference	
Under-expression	34	0.48 (0.29–0.79)		0.55 (0.33–0.90)	

Overall survival (OS), progression-free survival (PFS), hazard ratio (HR).

All significant factors were then incorporated into the prognostic model to develop individualized nomograms of OS at 3 and 5 years, and PFS at 3- and 5-years.

**Fig. 3:**
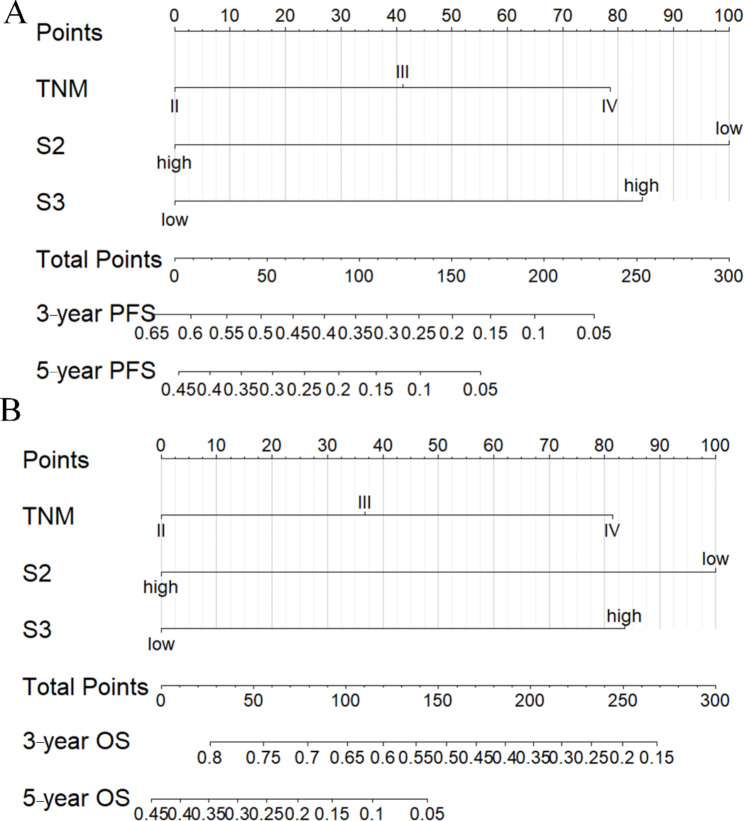
Nomograms for predicting survival analysis: (**A**) probability with 3- and 5-year OS; (**B**) probability with 3 and 5-year PFS.

As shown in the nomogram of OS (Fig. 3A), S2 presented the largest contribution to the prognosis, followed by TNM stages S1 and S3. Similarly, in the nomogram of PFS (Fig. 3B), S2 presented the largest contribution to the prognosis, followed by S1 and S3.

**Fig. 4:**
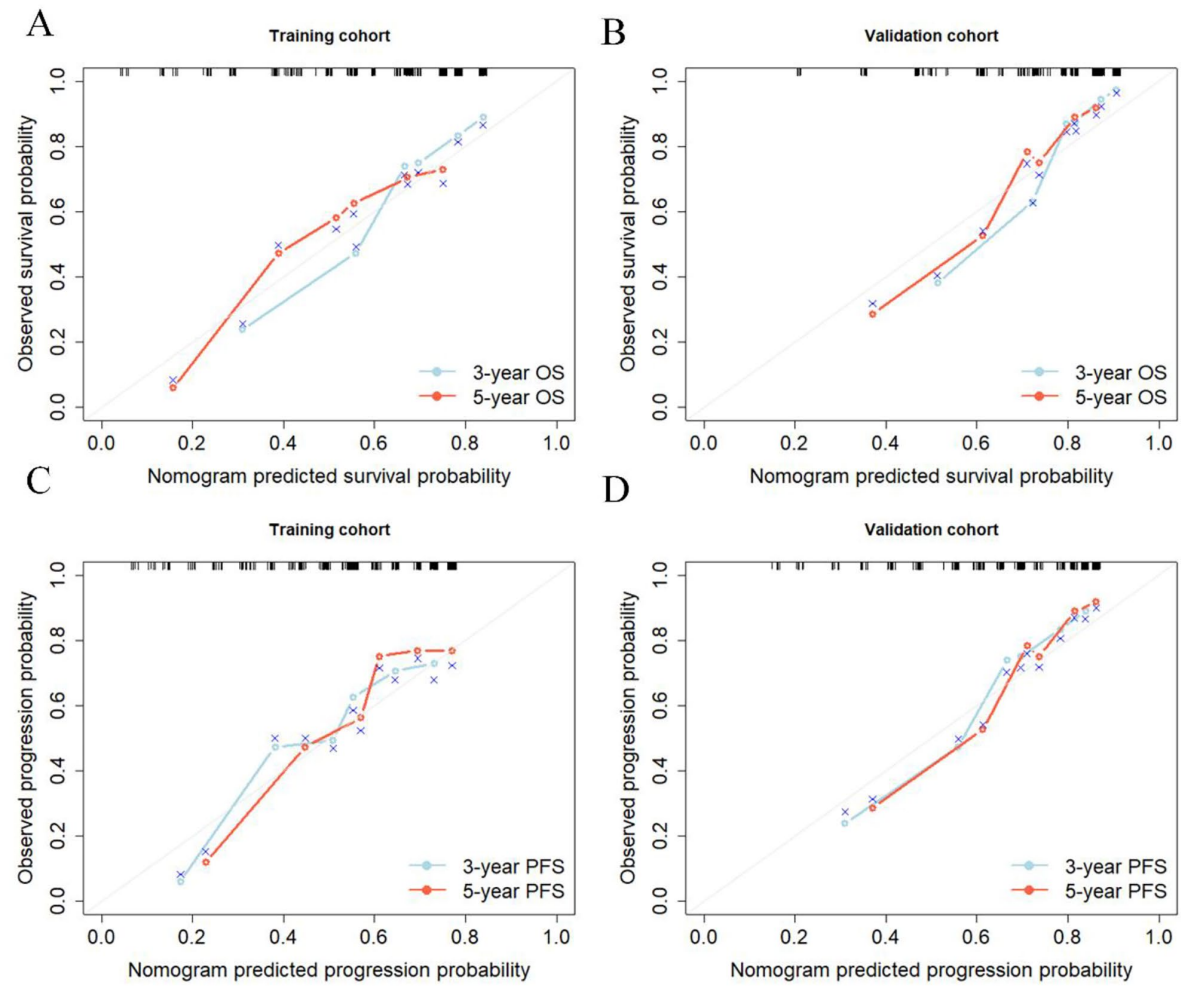
Calibration plots of the nomograms: (**A**) OS in the training cohort, (**B**) OS in the validation cohort, (**C**) PFS in the training cohort, (**D**) PFS in the validation cohort

The calibration curves **(**Fig. 4A and B**)** obtained from the individualized nomogram demonstrated a good consistency between the prediction and actual observation for both the 3-year and 5-year OS in the training and independent validation cohorts. Performances of the training and validation cohorts are shown on the plot relative to the 45-degree line, which represents a satisfied prediction. The mean absolute value for the 3-year and 5-year OS were 0.068 and 0.072 in the training cohorts, and 0.054 and 0.052 in the independent validation cohorts. The mean absolute value for the 3-year and 5-year PFS were 0.081 and 0.077 in the training cohorts, and 0.046 and 0.041 in the independent validation cohorts. The Harrell C-index (95% CI) of the nomogram was 0.74 (0.70–0.79) for the training cohort, and 0.72 (0.67–0.78) for the validation cohorts. For PFS in Fig. 4 C and 4D, the Harrell C-index (95% CI) of the nomogram was 0.71 (0.68–0.81) for the training cohort, and 0.72 (0.66–0.79) for the validation cohorts.

**Fig. 5:**
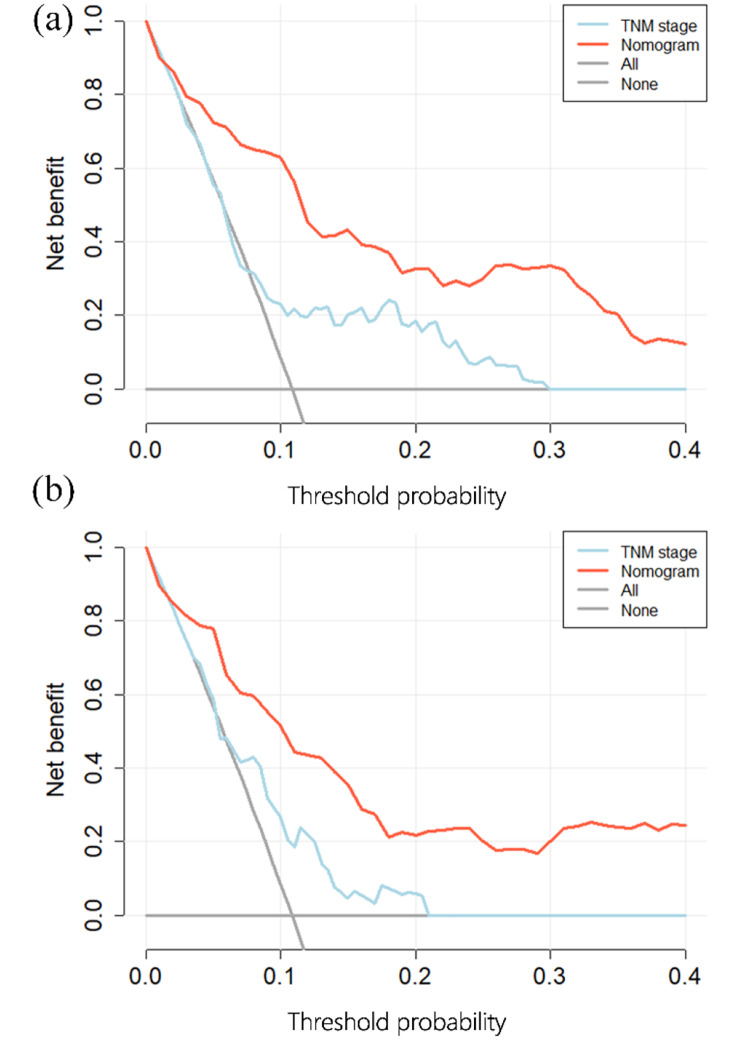
Decision curve analysis of nomogram and TNM stage in the validation cohort: (**A**) Decision curve analysis of OS; (**B**) Decision curve analysis of PFS.

As shown in Fig. 5, we compared the performance of the aforementioned models with that of the TNM stage using DCA. Our model provided the largest overall net benefit in predicting both OS and PFS compared to the TNM stage with (C-index (95% CI), OS:0.64 (0.58–0.69); PFS:0.62 (0.54–0.67)).

## Discussion

In this study, nomograms were established to predict the prognosis of NSCLC patients treated with chemotherapy as the first-line treatment. A total of 187 cases were included, and five significant prognostic factors representing histopathologic, radiomic, and deep learning features were identified by conducting univariate and multivariate analyses in the training cohort and then integrated to construct the nomogram. Integrated nomograms with three factors including the TNM stage, peritumoral radiomic signature, and tumoral cuboid-based deep learning signature were built according to the aforementioned factors.

To the best of our knowledge, this is the first study to combine clinical, radiomic, and deep learning features for predicting the survival of NSCLC patients treated with chemotherapy alone as the first-line treatment. Through this clinical calculator, both physicians and patients can easily predict individualized survival and inform patients regarding the individual benefits of chemotherapy. Patients can simultaneously be stratified into different risk subgroups to guide clinical decision-making. Among all the NSCLC patients, according to our nomograms, we should perform a more personalized plan of chemotherapy regimens and shorten the follow-up period for the high-risk subgroup.

For OS, the TNM stage, S2 and S3 remained as the significant prognostic factors, and S2 (HR (95%), 2.26 (1.40–3.67), p < 0.001)) outperformed the other three. This indicates that the hazard of under-repression in S2 was 2.26 times that of over-repression. The hazard of TNM stage III and IV was 1.48 and 2.10 times that of TNM stage II, respectively. The hazard of under-expression in S3 was 0.48 times that of over-expression. For PFS, TNM, S2 and S3 remained as the significant prognostic factors, and S2 (HR (95%), 2.23 (1.36–3.65); p < 0.05) outperformed the other two. The hazard of the underexpression subgroup was 2.23 times that of the over-expression in S2. The hazard of TNM stage III and IV was 1.42 and 1.98 times that of TNM stage II, respectively. The hazard of the under-expression subgroup in S3 was 0.55 times that of the over-expression.

For radiomics features, the peritumoral region performed better than the intratumoral region in terms of both OS and PFS. Patients with a high survival rate tended to have an overexpression signature in the peritumoral region, opposite to the intratumoral region (S1, Supplementary Fig. S2). This is likely because the homogeneity and heterogeneity of the two regions are opposite, and the heterogeneity both in and around the tumor is a significant predictor of survival in patients with NSCLC [33–36]. The prognosis of NSCLC is reflected in both the lesion and surrounding tissues [37–39]. Lymphatic canals present in peritumoral regions have a significantly higher overall survival rate in NSCLC [40]. A combination of peri- and intratumoral radiomic features can predict the treatment response in lung adenocarcinoma [41]. For deep-learning features, S3 had a significant prognosis for both OS and PFS. A flexible number of convolution kernels in AlexNet and fewer layers may be better for the small datasets used in this study.

Our results demonstrated that the nomograms represent a more precise prognostic prediction performance than that of the TNM staging system, which is consistent with the previous studies [42–44]. Xu et al. designed a nomogram to predict the OS of gastroenteropancreatic neuroendocrine tumors and demonstrated a better performance than the traditional TNM staging system [42]. Lin et al. proved that a nomogram with three risk factors performed better than the TNM stage system for OS in hepatoid adenocarcinoma of the stomach [43]. The TNM staging system considers anatomic features (depth of tumor invasion into the rectal wall, locoregional lymph node metastases, and distant metastases) but ignores the physical condition, pathological or genotype characteristics, and radiomic or deep learning factors [10, 44].

Many studies have applied the nomogram to fit patient survival curves to guide clinical treatment [45], but most studies currently apply clinical indicators combined with radiomic features or deep learning features [17, 19]. Because clinical features are readily available and easy to interpret for patients, they are widely used for survival analysis in clinical scenarios [46]. Radiomic features allow quantitative analysis of information about a lesion on a CT image to simulate a physician’s diagnosis of a patient and are also widely used in survival analysis [20, 47]. Deep learning features can extract high-dimensional image information that is invisible to the naked eye, allowing for deeper survival analysis [48, 49]. The innovation of this paper is to combine the information of the above three modalities and find the optimal combination of features to fit the patient’s survival curve through univariable and multivariable Cox analysis, so as to assist the doctor’s clinical diagnosis and treatment plan development.

The nomograms are limited by the retrospective nature of data collection and fail to incorporate certain recognized prognostic characteristics such as lymphatic permeation, vascular invasion, and molecular characteristics such as EGFR mutation and different chemotherapy drugs. We plan to improve the integrity of the data collection and patient follow-up in future studies. The data used in this experiment were obtained from a single center, which presents the disadvantage of generalization. We plan to collect more multicenter data for further verification, and a larger scale of patient populations is needed to identify potential risk factors.

## Conclusions

Nomograms integrating the TNM stage, peritumoral radiomic signature, and deep learning signature enable the prediction of individual prognosis measured by OS and PFS for NSCLC patients who received chemotherapy. The nomograms in this research provided a higher overall net benefit than the TNM stage in DCA curves and peritumoral radiomic and deep learning signatures outperformed the TNM staging system in predicting both OS and PFS. Being noninvasive and without the need for increasing additional expenses, signatures built from prechemotherapy CT images acquired in routine examinations have the potential to improve individualized treatment and precise management of patients with NSCLC.

### Electronic supplementary material

Below is the link to the electronic supplementary material.

Supplementary Material 1

## Data Availability

The datasets used and/or analyzed during the current study are available from the corresponding author on reasonable request.

## List of abbreviations

ADC: adenocarcinoma
C-index: Harrell’s concordance index
DCA: decision curve analysis
EGFR: epidermal growth factor receptor
HR: hazard ratio
KM: Kaplan-Meier
LASSO: least absolute shrinkage and selection operator
HR: hazard ratio
NSCLC: non-small cell lung cancer
OS: overall survival
PFS: progression-free survival
SCC: squamous cell carcinoma
SD: standard deviation
TNM: tumor, node, and metastasis
