# Supplementary material for: Nomograms integrating CT radiomic and deep learning signatures to predict overall survival and progression-free survival in NSCLC patients treated with chemotherapy
Source: Cancer Imaging. 2023 Oct 22;23:101. doi: 10.1186/s40644-023-00620-4 (PMC10590525; doi:10.1186/s40644-023-00620-4)
Supplement: Supplementary file 1 — Supplementary Material 1 [file 40644_2023_620_MOESM1_ESM.docx]

Supplementary Material for

**Nomograms Integrating CT Radiomic and Deep Learning Signatures to Predict Overall Survival and Progression-free Survival in NSCLC Patients with Chemotherapy**

## Fig. S1. Structure of deep learning models.

**
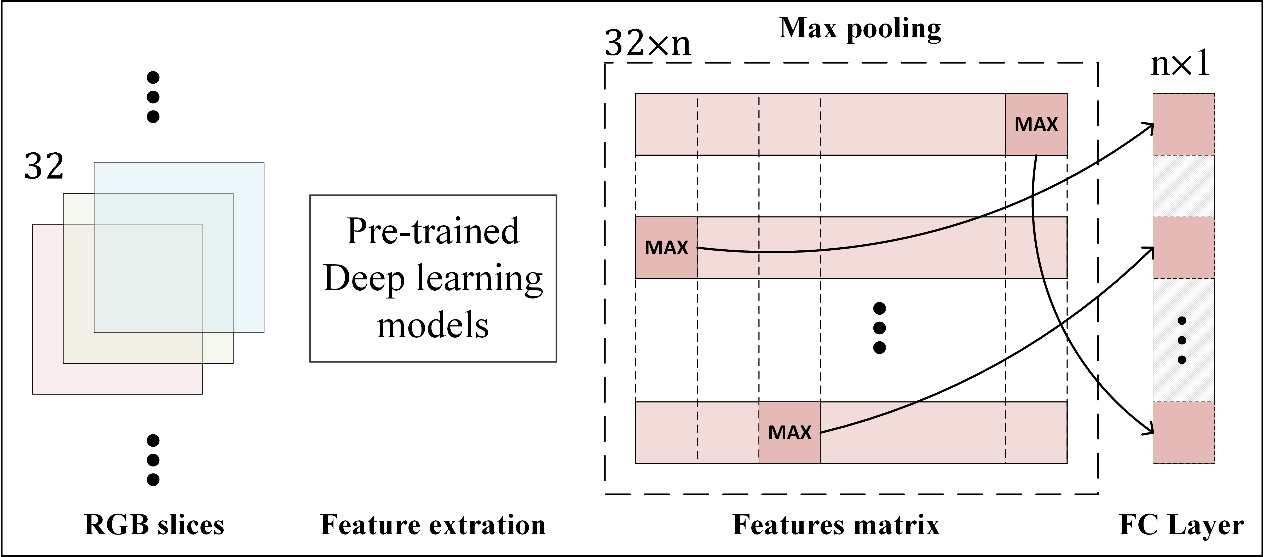
**

## Fig. S2. Kaplan-Meier curves of TNM stage for over- and under-expression subgroups: A, Kaplan-Meier curves in OS; B, Kaplan-Meier curves in PFS.

##
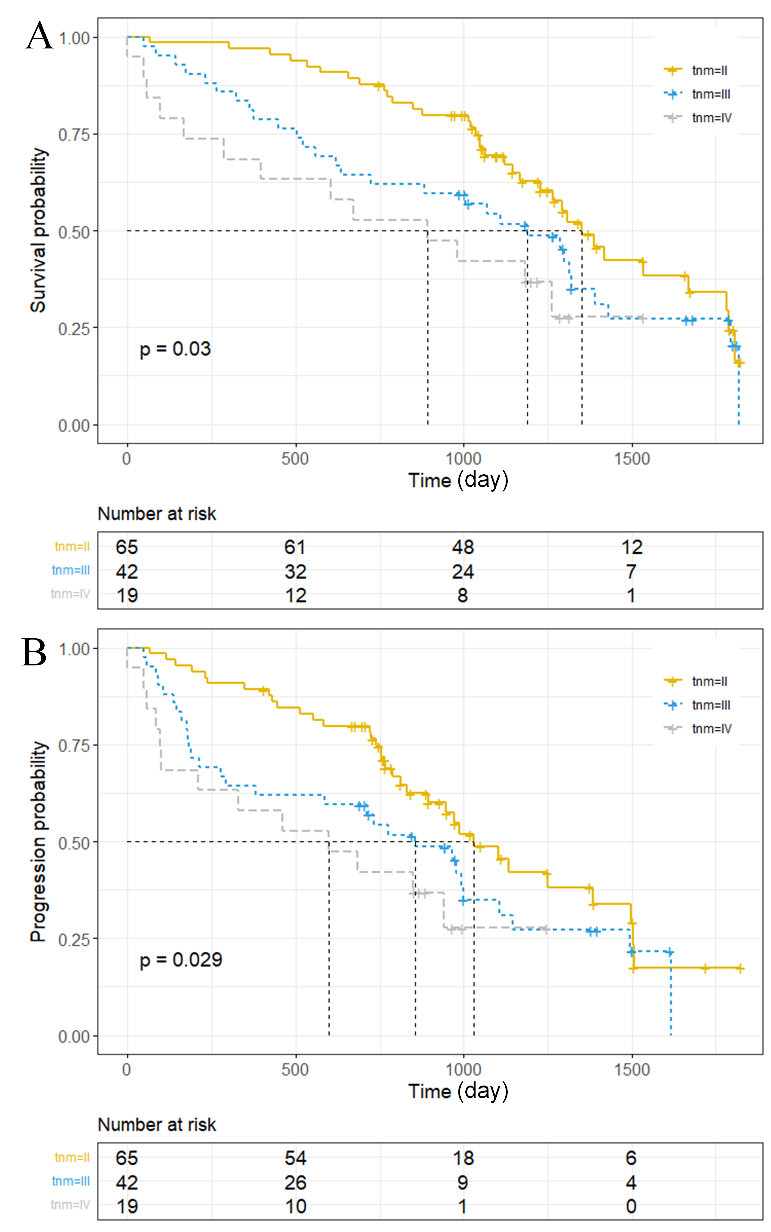


## Fig. S3. Kaplan-Meier curves of S1 for over- and under-expression subgroups: A, Kaplan-Meier curves in OS; B, Kaplan-Meier curves in PFS.

**
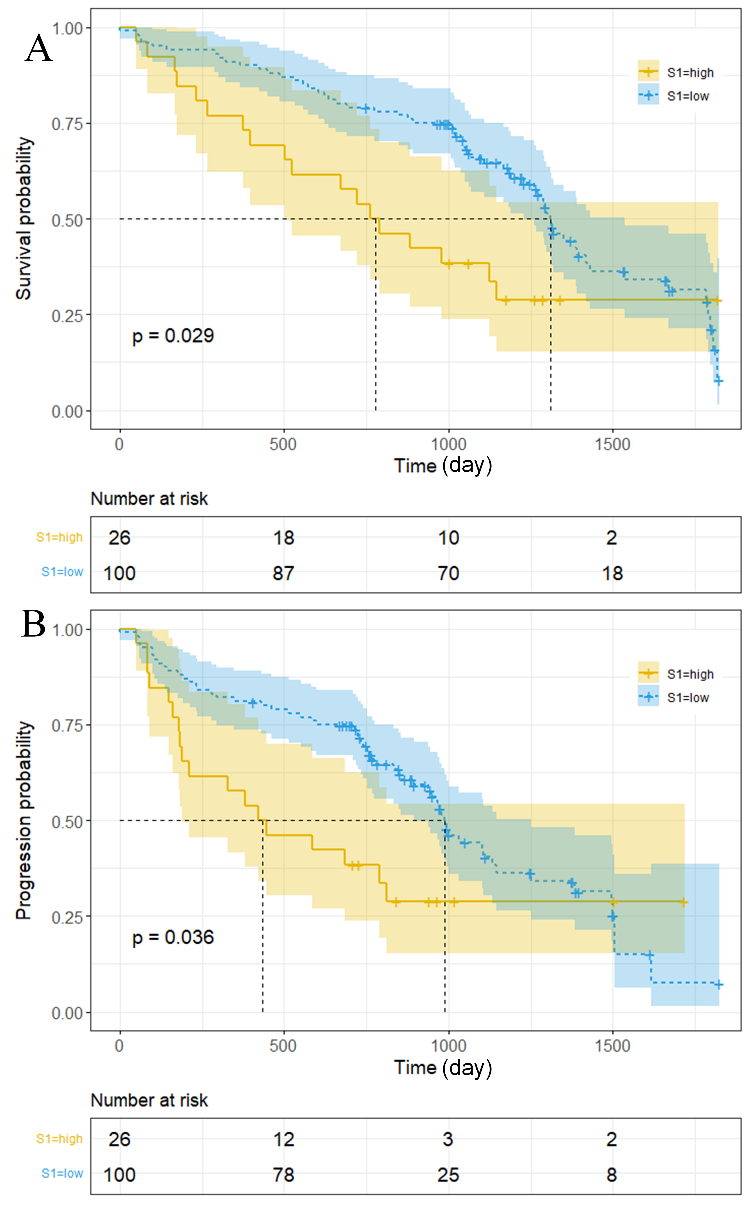
**

## Fig. S4. Kaplan-Meier curves of S3 for over- and under-expression subgroups: A, Kaplan-Meier curves in OS; B, Kaplan-Meier curves in PFS.

**
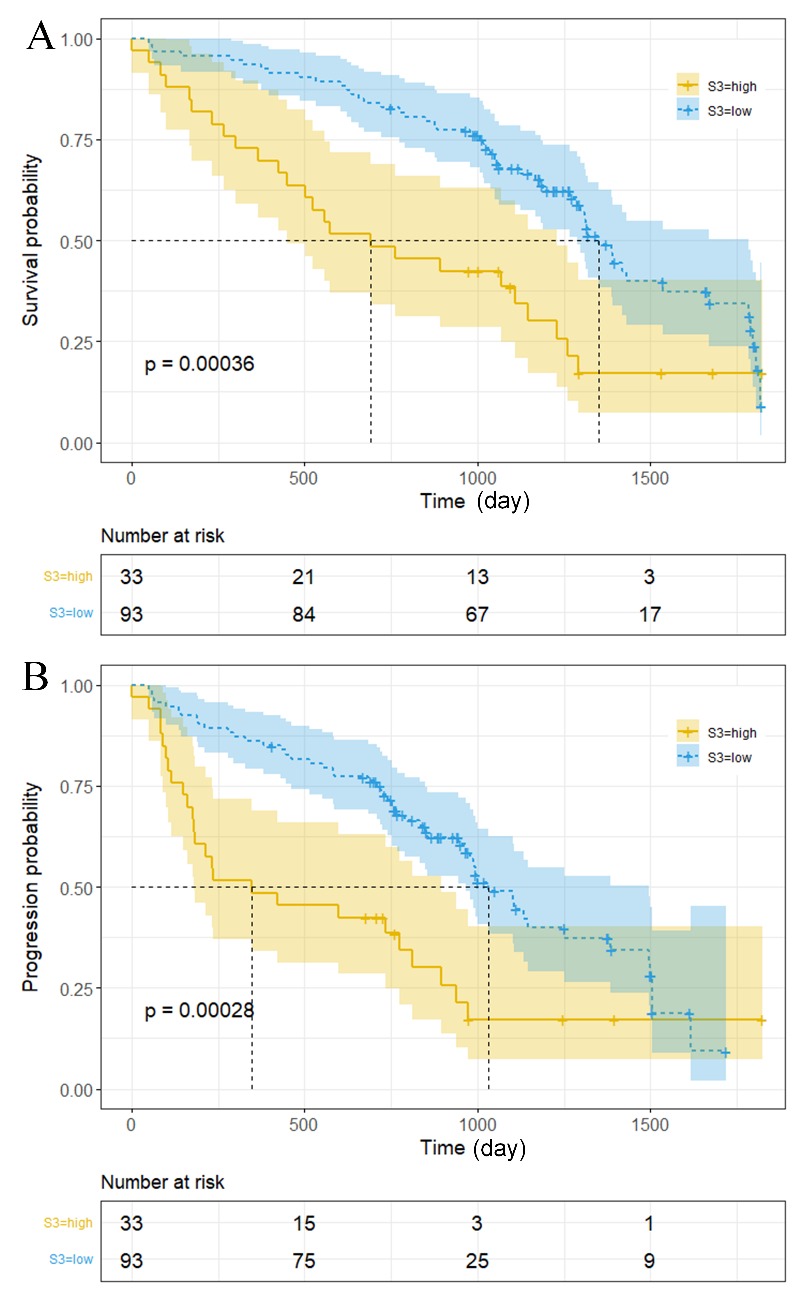
**
